# Gastro-Enterostomy

**Published:** 1918-08

**Authors:** 


**Gastro-Enterostomy.** C. W. Lippman of San Francisco, Calif. State Jour. of Med., Jan. 1918, analyses 100 cases: 17 required re-operation, 3 for return of ulcers, one gastro-jejunal and two in the stomach itself, 6 for adhesions found, 5 for symptoms supposed to signify adhesions but in which none were found, 3 for too large or relaxed stoma; 12 more were not relieved though further operation was not indicated, making 29 bad results. 8 cases resulted fatally, 12 could not be followed up and 51 gave good results. The last included 12 of stenosis of the pylorus for which gastro-enterostomy is a logical operation granting that the lesion itself cannot be directly attacked. So far as peptic ulcer is concerned, there were 39 successful and 37 unsuccessful cases. Of the 8 fatal cases, 4 were moribund at the time of operation, 1 each died of haemorrhage and of peritonitis, 2 of shock and 4 of pneumonia.

